# Potentially Bioaccessible Phenolics from Mung Bean and Adzuki Bean Sprouts Enriched with Probiotic—Antioxidant Properties and Effect on the Motility and Survival of AGS Human Gastric Carcinoma Cells

**DOI:** 10.3390/molecules25132963

**Published:** 2020-06-28

**Authors:** Michał Świeca, Anna Herok, Katarzyna Piwowarczyk, Małgorzata Sikora, Patryk Ostanek, Urszula Gawlik-Dziki, Ireneusz Kapusta, Jarosław Czyż

**Affiliations:** 1 Department of Biochemistry and Food Chemistry, University of Life Sciences, Skromna Str. 8, 20-704 Lublin, Poland; malgorzata.sikora@up.lublin.pl (M.S.); patryk.ostanek@gmail.com (P.O.); urszula.gawlik@up.lublin.pl (U.G.-D.); 2 Department of Cell Biology, Jagiellonian University, Gronostajowa Str. 7, 30-387 Cracow, Poland; anna.herok@gmail.com (A.H.); katarzyna.szpak@uj.edu.pl (K.P.); jarek.czyz@uj.edu.pl (J.C.); 3 Department of Food Technology and Human Nutrition, Rzeszów University, 4 Zelwerowicza Street, 35-601 Rzeszów, Poland; ikapusta@univ.rzeszow.pl

**Keywords:** AGS gastric cancer, phenolics, mung bean, adzuki bean, probiotic, sprouts, digestion in vitro

## Abstract

Gastric digests from mung (MBS) and adzuki (ABS) bean sprouts enriched with probiotic *Lactobacillus plantarum* 299v were tested for their antioxidant potential, as well as antiproliferative and antimotility properties, in human stomach cancer cells (AGS). The digest of ABS contained quercetin and kaempferol derivates, while kaempferol and apigenin derivates were dominant in MBS. Compared to the controls, the probiotic-rich sprouts had a higher antioxidant potential—by 13% and 9%, respectively. Adzuki bean sprouts decreased the viability of AGS already at low concentrations (25% motility inhibitions). MBS and ABS displayed dose-independent cytostatic effects. The ABS extracts decreased the proliferation of AGS more effectively than the MBS extracts—0.2‰ ABS exerted c.a. 70% of inhibitions. Moreover, the phytochemicals from the probiotic-rich sprouts considerably reduced this activity. The increased vinculin level, the apoptotic shape of cell nuclei, and the reduced cell motility and proliferation indicate that the extracts exhibited cytostatic and cytotoxic activity.

## 1. Introduction

Although the incidence of gastric cancer varies greatly across countries, this tumor is the fifth most common malignancy and the third leading cause of cancer-related mortality [1]. It is associated with environmental and genetic predisposition factors, including an unhealthy diet and lifestyle (e.g., high-salt food, smoking, and drinking). There is strong evidence that an increased intake of a diet rich in vegetables and fresh fruits may reduce the incidence of this disease. 

Numerous studies have confirmed that naturally occurring dietary phenolics have antiproliferative properties and are able to inhibit tumor angiogenesis and metastasis; therefore, these compounds are regularly referred to as anticancer agents. Experimental and clinical data show that phenolics may induce apoptosis, maintain the redox status of cells, downregulate the expression of cyclins and proinflammatory genes (e.g., cyclooxygenase-2 and NF-κB), upregulate the expression of MIC-1 and proapoptotic kinase genes, or exhibit direct toxicity against highly proliferating cancer cells [2,3]. In vivo anticancer activity strongly depends on the bioaccessibility, bioavailability, and metabolic fate of phenolics. They are usually absorbed through passive diffusion or mediated uptake into the intestine epithelial wall and are, finally, metabolized by phase I and II enzymes (e.g., CYPs, UGTs, and SULTs) into various metabolites [4]. However, in some cases (e.g., digestive tract cancers), phenolics may be directly absorbed by cancer cells and exert side effects [5].

Legume sprouts, including adzuki and mung bean, are excellent sources of nutrient and phenolic compounds [6]. There is also ample evidence that these compounds are responsible for the pro-health properties of food, e.g., anticancer and anti-inflammatory activities [7,8]. Additionally, it has been proved that bean sprouts may be effective carriers for probiotics, including *Lactobacillus plantarum* or *Saccharomyces boulardii* [9,10]. These microorganisms can be effectively employed for reduction of the growth of *Helicobacter pylori* (a factor positively correlated with gastric cancer) [11,12], the alleviation of some digestive tract disorders [13], the inhibition of autoaggressive and allergic reactions [14], or the improvement of the overall immunity of the human organism [15]. The presence of probiotics in sprouted food improves its microbiological quality [9,16] and, usually, causes positive changes in the phenolic fraction by induction of the phenylpropanoids pathway [17].

The aim of the study was to investigate the antiproliferative effects and the ability to reduce the motility of human stomach cancer cells (AGS) by fractions obtained after the gastric digestion of mung bean and adzuki bean sprouts enriched with probiotic *L. plantarum* 299v. Special emphasis was placed on the phenolic fractions and antioxidant capacities of the extracts.

## 2. Results and Discussion

Studies of the anticancer properties of food or its components are usually limited to the application of extracts, which does not reflect their real composition and bioactivity, as such results are often overestimated due to the use of highly efficient extraction systems. Such analyses omit potentially bioaccessible fractions that exhibit activities directly in the lumen of the digestive tract and/or may be effectively absorbed. In this study, a double strategy was employed for designing new functional products with improved anticancer properties, i.e., a combination of legumes (a source of phenolics exhibiting anticancer properties) with probiotic *L. plantarum* 299v (limiting gastric disorders, including inflammation caused by *H. pylori* [11]). However, these properties of probiotics are difficult to confirm in in vitro systems. It can be expected that they will be able to support activities exhibited by the bioactive components of probiotic-rich sprouts in vivo. Many reports have shown that the anticancer properties of legumes are strongly associated with bioactive components such as phenolics, proteinase inhibitors, or lectins [18]. As demonstrated in the literature and our previous studies, the content of lectins and protease inhibitors is marginal and significantly reduced in sprouts, compared to dormant seeds [19,20]. Hence, it may be speculated that a key role in the cytostatic effects of the studied gastric digests is played by phenolics compounds.

To correlate the anticancer potential of gastric digests with the phenolic contents and compositions, detailed analyses of these compounds were performed. The phenolic contents in the fractions obtained after the simulated gastric digestion of the control and probiotic-rich adzuki bean and mung bean sprouts are presented in Table 1. Thirteen different phenolics were identified in the fraction obtained from the adzuki bean sprouts. These were mainly quercetin and kaempferol derivates, especially quercetin 3.4′-*O*-diglucoside and quercetin 3-*O*-rutinoside (rutin). Compared to the probiotic-rich sprouts, the control sprouts contained more phenolics (by about 5%); however, the differences were not significant. The mung bean sprouts contained kaempferol and apigenin derivates. The dominant phenolics in the studied fractions were apigenin 3-*O*-glucoside—its contents ranged from 323 µg/g in the control to 364 µg/g in the *L. plantarum*-enriched sprouts. Most importantly, the probiotic-rich sprouts contained about 18% more phenolics than the control. 

Zhang et al. [21] suggested that the anticancer properties of phenolics, including apigenin, kaempferol, and quercetin, were often related to their antioxidant properties, such as the ability to quench free radicals and chelate transition metal ions (substrates of the Fenton reaction), lipid-protective properties, or reducing the potential. There was no impact of *L. plantarum* on the ability to neutralize the ABTS radical; however, a higher activity was found for the adzuki bean sprouts (Table 2).

The physiological radicals (OH^•^ and O_2_^−^) were more effectively neutralized by mung bean sprouts. In the case of the adzuki bean, the potentially bioaccessible fractions from the control sprouts were more effective quenchers of the superoxide anion. Most importantly, a positive effect of the probiotic was clearly visible in the mung bean sprouts, i.e., an increase by 19% and 30% for the superoxide anion and hydroxyl radicals, respectively, was observed. Such a phenomenon was also observed in the case of the reducing power and ability to chelate metal ions, as there was an increase by about 7% and 39%, respectively. Generally, in both adzuki and mung bean sprouts, better results were found for the probiotic-rich sprouts, i.e., an increase in the total antioxidant index by 13% and 9%, respectively.

The qualitative composition of the mung bean sprouts was in the agreement with previous studies conducted by Tang et al. [22], indicating that apigenin and kaempferol glucosides were the dominant phenolics. Similarly, Bai et al. [23] reported that mature seeds of the adzuki bean contained mainly kaempferol glucosides, rutin, and anthocyanins. The fraction obtained after gastric digestion contained both kaempferol and quercetin glucosides; however, no anthocyanins were detected. These phenolics are usually lost during the soaking of seeds before sprouting [19], and their extractions to the digests can also be limited by their interactions with the nutrients (protein, starch, and lipids) and digestive enzymes [24] and by restructuration under variable pH conditions and/or due to the actions of the enzymes [25]. However, a direct quantitative comparison of the results is difficult due to many factors (sprouting conditions, extraction system, bean variety, etc.). In previous studies, the antioxidant properties of samples were positively correlated with the phenolic content. In turn, the gastric digest of probiotic-rich sprouts had slightly higher antioxidant properties. In previous studies of plant extracts, e.g., for gastric cancer treatment, the cytotoxic effects were linked with the content and antioxidant properties of phenolics [3,26].

**Figure 1:**
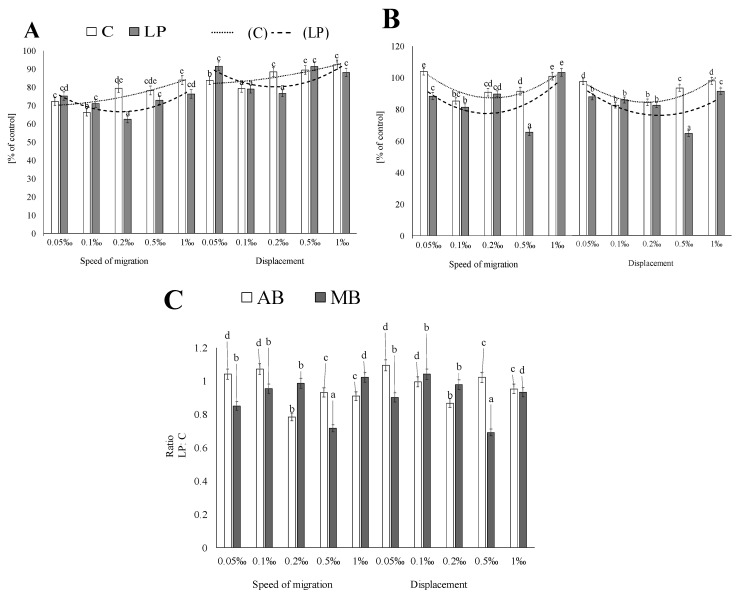
Effects of the digest on the speed and migration of human gastric cancer (AGS) cells. (**A**) Adzuki bean (AB), (**B**) mung bean (MB), and (**C**) comparison control vs. probiotic-rich sprouts. Bar graphs depict averaged AGS motility and displacements estimated from the trajectories of at least 50 cells (control = 100%). Means (±SD) for the selected activity followed by different letters are significantly different (*n*= 9; *p* ≤ 0.05). C—control sprouts and LP—sprouts enriched with *Lactobacillus plantarum.*

**Figure 2:**
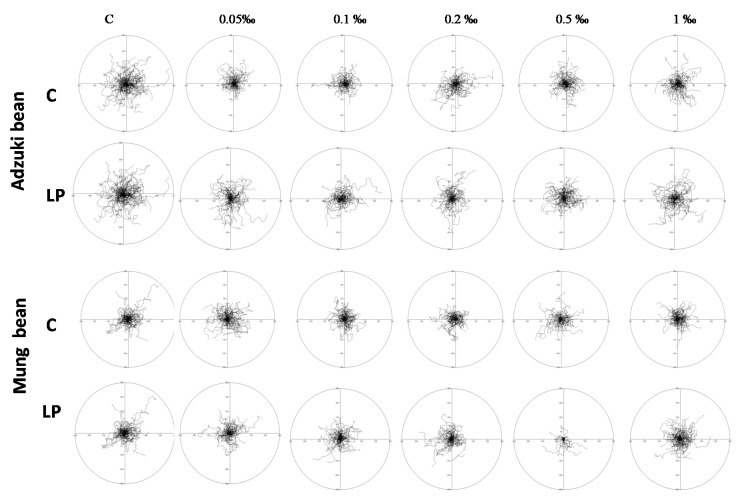
Effects of the control (C) and *Lactobacillus plantarum*-fortified sprout extracts on the motility of human gastric cancer (AGS) cells. Circular diagrams show cell trajectories of the cells incubated with the studied extracts.

To assess the short-term effects of potentially bioaccessible phenolics from the sprout extracts on AGS viability, extract-supplemented media were applied to the AGS cell cultures, and cell trajectories were traced immediately afterwards for 8 h (Figure 1 and Figure 2). The adzuki bean sprout extracts exerted short-term inhibitory effects on AGS viability already at low concentrations, i.e., ca. 25% inhibition of AGS motility was observed in the presence of the 0.05‰ extract from both the control and probiotic-rich sprouts. The potentially bioaccessible compounds from the probiotic-rich adzuki bean sprouts exerted dose-independent cytostatic effects on AGS motility (Figure 2). This was illustrated by the relatively strong inhibition of AGS motility observed at the low extract concentration (0.05‰) and the comparable cytostatic effect observed when the extract was applied at the higher concentrations (0.5–0.1‰)—the response to the studied concentrations of extracts may be described by a U-shaped curve. The cytostatic activity of the compounds from the control adzuki bean sprouts was hardly dose-dependent; actually, a slight decrease in the cytostatic activity was seen in the presence of the higher extract concentrations (Figure 1A). In turn, the mung sprout extract exerted rather negligible short-term cytostatic effects on the AGS cells in the range of the concentrations applied. The highest inhibition of AGS motility was recorded for the 0.5‰ extract of the probiotic-rich sprouts. As in the case of the probiotic-rich adzuki bean extracts, the mung bean extract also exerted dose-independent cytostatic effects (Figure 1B). In fact, there was no effect at the lowest and highest concentrations studied, while extracts with a concentration range from 0.1‰ to 0.5‰ had significant cytostatic properties. Comparing the results obtained for the control and probiotic-rich sprouts, it was visible that the coculture enhanced the antimotility properties of the sprouts (Figure 1C). This was especially clearly visible when the adzuki bean extracts were applied at the higher concentrations (0.2–1‰ and 1.0‰ in the case of the mung bean extracts). 

**Figure 3:**
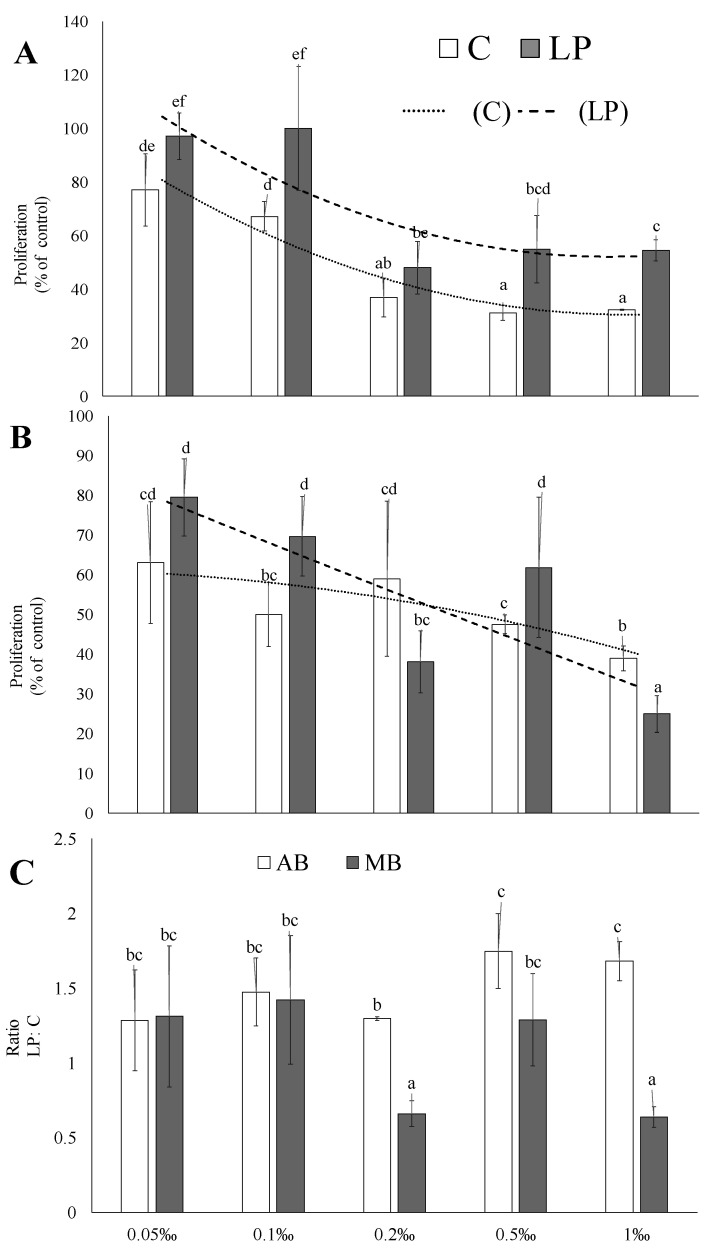
Effects of the digest on the proliferation of the AGS line. (**A**) Adzuki bean sprouts, (**B**) mung bean sprouts, and (**C**) the probiotic-rich sprouts vs. the control. Bar graphs depict the averaged AGS proliferation (control = 100%). Means (± SD) for the selected activity followed by different letters are significantly different (*n* = 9; *p*≤ 0.05). C—control sprouts and LP—sprouts enriched with *Lactobacillus plantarum*.

**Figure 4:**
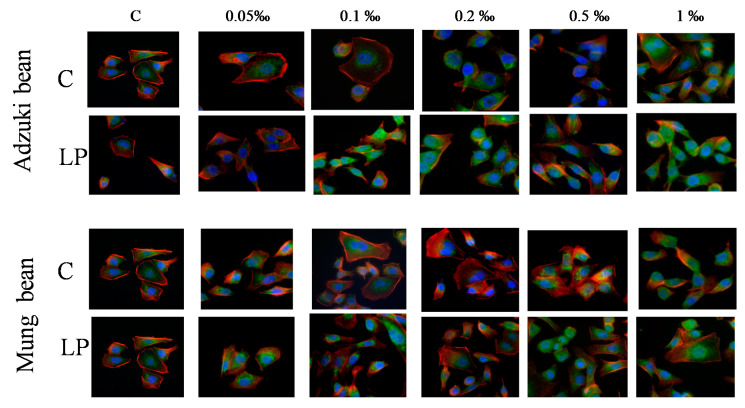
Effects of the control (C) and *L. plantarum*-fortified sprout extracts (LP) on the actin cytoskeleton architecture of human gastric cancer (AGS) cells. Actin—red, vinculin—green, and DNA—blue.

The quality and quantity of the long-term AGS reactions to the adzuki bean sprout extracts were more prominent than those observed in the presence of the mung bean sprout extracts (Figure 3). Probiotics considerably reduced this activity, especially when the extracts were administered at the high concentrations (Figure 3A). Moreover, the extracts of the probiotic-rich sprouts administered at the 0.05‰ and 0.1‰ concentrations did not have a cytostatic effect. The highly concentrated extracts from the control adzuki bean sprouts (0.2–1‰) exerted a high cytostatic effect, i.e., ca. a 70% proliferation inhibition (Figure 3A). A less prominent (70% inhibition was reached only by the 1‰ extract) but linear cytoprotective effect was seen in the presence of the mung sprout extract (Figure 3B). At the lower concentrations, extracts from the control sprouts were more effective; however, a positive effect of the probiotic was visible when the 0.2‰ and 0.1‰ extracts were tested (Figure 3C). A morphological analysis of the AGS cells (data not shown) showed that the 0.5‰ and 1‰ extracts caused a reduction of cell flattening compared to the untreated lines. After Hoechst 33342 staining, the cell nuclei of the AGS cells treated with the high concentrations of the extracts were uniformly round or oval. A similar observation was reported by Chen et al. [27], where the cell nuclei of HGC-27 and SGC-7901 cells after an apigenin treatment exhibited the characteristics of apoptosis. Such nuclear morphological changes in AGS cells were also recorded by Zou and Chang [8] after the application of a black soybean extract. The cells treated with the extract obtained from probiotic-rich sprouts displayed an increase in the vinculin level, which may indicate that the extracts exhibit both cytostatic and cytotoxic activity (Figure 4).

Both apigenin and quercetin (the main phenolics determined in the gastric digest of both the control and probiotic-rich sprouts) can affect the cell signal transduction and gene expression, as well as the metabolism, proliferation, invasion, and differentiation of tumor cells [28,29]. Data obtained by Chen et al. [27] showed that apigenin broke the balance of the Bcl-2 and Bax proteins, promoting the action of the proapoptotic Bax protein. Importantly, there was no negative impact on the normal gastric epithelial cell line GES1 treated with different concentrations of apigenin [27]. Quercetin also decreased the Bcl-2/Bax ratio subsequently, causing an increased expression of caspase-3 [29]. These observations suggest that apoptosis may be mediated via the mitochondrial pathway. On the other hand, by interrupting the intracellular redox balance (the production and detoxification of the reactive oxygen species (ROS)/RNS), phenolic antioxidants influence the signal transduction and regulation of the cell cycle (progression from the G0/G1 to the S and G2 to the M phases). Such a mechanism may be very effective in proliferative diseases like cancer [28]. Phenolics inhibit cell proliferation, causing direct damage to cellular components, i.e., membranes, ion channels, and protein, and promote apoptosis; however, the mechanisms of action usually overlap [3,8,27,29]. Usually, the range of concentrations tested in in vitro tests is far beyond ranges that can be achieved in humans for disease prevention or therapy. In our study, we tried to confirm that a phenolic-rich extract may effectively influence cancer cells at physiological concentrations. The highest concentrations of the phenolics (1‰) tested in this study were approx. 400 nM and 1.3 µM for adzuki bean and mung bean sprouts (calculated based on quercetin and apigenin), respectively. These values are in the range of biological concentrations of trans-resveratrol (120–150 nM) [30], quercetin (1 µM) [29], and epigallocatechin gallate (51–72 nM) observed in an in vivo human model [30].

So far, phenolics have proven to be effective in gastric cancer treatment; however, the mode of their action was different or unclear. Phenolics from *Alnus japonica* ethanol extracts inhibited the growth of AGS human gastric carcinoma cells and induced cell death by increasing the production of the reactive oxygen species (ROS) in AGS cells in a dose- and time-dependent manner [26]. Generally, the extracts acted in a dose- and time-dependent manner; however, at the lowest concentration (c.a. 18 µg of flavonoids per mL of the medium), an increase in the growth of SNU-1967 and SNU-601 human gastric carcinoma cells was recorded after 12 h of treatment. A similar behavior was observed in our study of the effect of mung bean extracts on the rate and intensity of the migration of AGS cells, where the lowest and the highest concentrations studied did not cause a desirable decrease in the analyzed parameters. Xu and Chang [31] showed that lyophilized hydrophilic extracts of mung and adzuki beans showed dose-dependent inhibitions against gastric adenocarcinoma cells (AGS) after 48-hr applications in the range of 0–5 mg/mL, with IC_50_ of 1.02 and 0.68 mg of phenolics/mL, respectively. Similar observations were found in the cases of the gastric digests of both the control and probiotic-rich mung bean sprouts. In the study conducted by Xu and Chang [31], some extracts exerted a dose-independent effect on the proliferation of the studied cell lines. Compared to the untreated samples, the addition of low-concentrated mung and adzuki bean extracts (0.05–0.1‰) improved the proliferation of Caco-2 cells. The viability of colon cancer cells decreased rapidly only in the range of 0.25–1 mg/mL of the adzuki bean extract, while the effect was constant after the application of higher concentrations. Similarly, the adzuki bean extracts in our study worked in a dose-dependent manner only in the concentration range from 20 to 80 nM of the phenolics (0.05–0.2‰), while the further increased phenolic load did not cause a predicted effect. A dose-independent effect was also observed after the application of extracts from legumes, e.g., yellow pea, yellow soybean, and black-eyed pea to the AGS line or black-eyed pea to colorectal adenocarcinoma cells SW 480 [31]. Both the level of oxidative DNA damage and intracellular ROS were significantly affected by concentrations of an anthocyanin-rich bilberry extract used in human colon tumor cell lines Caco-2 and HT-29 [32]. Surprisingly, at a concentration range of 1–50 µg/mL, the ROS level was significantly higher than in the control. A reverse effect was found for oxidative DNA damage; concentration curves resembling a “U-shape” reaching a minimum at 5 µg/mL were observed. As mentioned before, a similar phenomenon was found in our study after the application of the mung bean extracts. It may be suggested that low concentrations of phenolics protect cancer cells against oxidative damage (phenolics are “directly” utilized in the redox system). Such an explanation may be supported by a previous study of black soybean and *Alnus japonica* extracts on the AGS cell viability [8,26]. Typically, the desirable cell death in different gastrointestinal tract cancers is usually observed at a higher concentration range.

## 3. Materials and Methods 

### 3.1. Materials

All chemicals used for the cultivation of sprouts and microbiological media were purchased from the SigmaAldrich company (Poznan, Poland) and BTL Ltd. (Łodz, Poland). Adzuki bean and mung bean seeds were purchased from the PNOS S.A. in Ozarów Mazowiecki, Poland. Strain *L. plantarum* DMS 9843 (299v) was used for the coculture [9].

### 3.2. Sprouting Conditions

The seeds were disinfected in 1% (*v*/*v*) sodium hypochlorite for 10 min, drained, and washed with distilled water until they reached a neutral pH. Next, they were soaked in distilled water (C, control) or a Lactobacillus plantarum DMS 9843 (299V) water suspension (1 × 10^8^ CFU per 1 g of seeds), (LP, enriched with probiotics). The adzuki and mung bean seeds were soaked for 6 and 8 h, respectively. The seeds (approximately 12 g per plate) were dark-germinated for 4 days in a growth chamber (MLR-350H, Sanyo, Japan) on Petri dishes (φ 125 mm) lined with absorbent paper (relative humidity 90%). Seedlings were sprayed daily with 5 mL of Milli-Q water. Sprouting was carried out at 25 °C. Four-day-old sprouts were manually collected and immediately frozen, freeze-dried, and stored in polypropylene boxes at −60 °C [9].

### 3.3. In Vitro Digestion

In vitro digestion was performed as described previously by [33]. After digestion, the samples were centrifuged (15 min 6900× *g*), immediately frozen, freeze-dried, and stored at −20°C. For the test, the samples were dissolved in PBS buffer (phenolics and antioxidant tests) or in the culture medium (anticancer tests).

### 3.4. Phenolic and Antioxidant Test

#### 3.4.1. Phenolic Assay

The crude extract was passed through a C18 Sep-Pak (360 mg, 55–105 μm) cartridge (Waters Associates, Milford, MA, USA) preconditioned with water. The cartridge was washed first with water (10 mL) to remove sugars and, then, with MeOH (10 mL) to elute phenolics. This fraction was evaporated to dryness and redissolved in 50% MeOH for analyses. 

Structural information and general phenolic profiles were gathered using a Waters Acquity UPLC system (Milford, MA, USA) consisting of a binary solvent manager, a sample manager, a PDA detector, and a triple-quadrupole detector (TQD) operating in the negative electrospray mode [34]. 

#### 3.4.2. Antioxidant Capacity

##### Reducing Power

Reducing power was determined with the method of Pulido, Bravo, and Saura-Calixto [35]. Reducing power was expressed as Trolox equivalents in mg per g of dried sprouts.

##### Inhibition of Lipid Peroxidation

The antioxidant activity was determined as the degree of inhibition of the hemoglobin-catalyzed peroxidation of linoleic acid according to Goupy et al. [36]. Inhibition of linoleic acid peroxidation was expressed as Trolox equivalents (TE) in mg per g of dried sprouts. 

##### Ability to Chelate Metal Ions

The chelating power was determined with the method of Decker and Welch [37]. 

The chelating power was expressed as EDTA equivalents (EDTA) in mg per g of dried sprouts.

##### Ability to Quench ABTS Radicals

The experiments were carried out using the ABTS decolorization assay [38]. The free radical scavenging ability was expressed as Trolox equivalents in mg per g of dried sprouts.

##### Ability to Quench Hydroxyl (OH^•^) Radicals

The OH^•^ scavenging ability was determined according to [39]. The free radical scavenging ability was expressed as Trolox equivalents in mg per g of dried sprouts.

##### Ability to Quench Superoxide Anion Radicals (O_2_^–^)

The ability to quench O_2_^–^ was determined according to a procedure described by [39]. The free radical scavenging ability was expressed as Trolox equivalents in mg per g of dried sprouts.

##### Total Antioxidant Activity Index (AI)

All complementary antioxidant methods were integrated to obtain the total antioxidant activity index (AI) [40]. The index can be useful for the evaluation of the total antioxidant potential of modified foods in respect to the control. The AI was calculated as the sum of relative activities (RA) for each antioxidant chemical methods divided by the number of methods.

(1) $$IA=\frac{\Sigma^{}RA_{\left(n\right)}}{n}$$

The RA was calculated as follows:

(2) $$RA=\frac{A_{x}}{A_{c}},$$

where $A_{x}$—activity of the modified food and $A_{c}$—activity of the control.

### 3.5. Effect on Human Stomach Cancer AGS and Human Colon Carcinoma HT-29 Cells

#### 3.5.1. Culture Conditions

Analyses of the biological activity of the extracts were carried out using human stomach cancer (AGS) cells (number 89090402, Sigma, Poznan, Poland). Cells were routinely cultivated in RPMI medium supplemented with 10% FBS and antibiotics in a humidified 37 °C/5% CO_2_ atmosphere. Lyophilized extracts from the mung and adzuki bean sprouts were dissolved in the culture medium in the proportion of 1:100 (*w*/*v*). The stock solutions were then dissolved in the culture media to reach the final concentrations between 0.05‰ and 1‰, which was equivalent to the range of concentrations relevant for the gastric environment.

#### 3.5.2. Proliferation Tests

Long-term (up to 72 h) effects of the extracts on AGS cells were estimated with Coulter Counter-assisted proliferation tests. For the assay, AGS cells were seeded into 12-well flasks (Nunclon, Sigma, Poznan, Poland) at an initial density of 7.5 × 10^3^ cells/cm^2^ and cultivated for 24 h. Next, the culture medium (RPMI supplemented with 10% fetal bovine serum, all from Sigma, Poznan, Poland ) was exchanged or replaced with medium containing the extracts (administered from stock solutions to reach the final concentrations indicated in the text). The cells were cultured for the next 72 h and counted with a Coulter counter. 

#### 3.5.3. Motility Tests

Time-lapse videomicroscopy studies of AGS cell motility were performed on cells plated into 12-well flasks (Corning, Sigma, Poznan, Poland) at initial cell densities chosen to compensate for the inhibitory actions of the extracts on the cell proliferation (200 to 400 cells/mm^2^). Supplemented media were applied to the cells immediately before the registration of their movements to estimate the short-term (up to 8 h) effects of the extracts on the AGS viability. The movement of individual cells was recorded immediately after the extract administration along with the culture medium (RPMI, Sigma; see above) with the computer-assisted Leica DMI6000B system (Hayward, CA, United States) (recording time: 8 h with 5-min time intervals at 37 °C). Cell trajectories (N > 50 cells, three independent experiments) were pooled and statistically analyzed. The following parameters were estimated: (i) the total length of cell displacement (TLCD; μm), i.e., the distance from the starting point directly to the cell′s final position, and (ii) the average speed of cell movement (Speed of migration; μm/min), i.e., the total length of the cell trajectory/time of recording (8 h) [41].

#### 3.5.4. Cytoskeleton Architecture

The cytoskeleton architecture of the AGS cells was analyzed in formaldehyde-fixed Triton-solubilized cells stained with rabbit anti-vinculin IgG (number V9131, Sigma, Poznan, Poland) and counterstained with Alexa 488-conjugated goat anti-rabbit IgG (number A11008, Invitrogen, Warsaw, Poland), TRITC-conjugated phalloidin (number 77418, Sigma, Poznan, Poland), and Hoechst 33258 (number 861405 Sigma, Poznan, Poland). The image acquisition was performed with a Leica DMI6000B microscope (Leica Microsystems, Wetzlar, Germany) equipped with the total internal reflection fluorescence (TIRF) and interference modulation contrast (IMC) modules.

### 3.6. Statistical Analysis

All experimental results are means ± SD of three parallel experiments. Two-way analysis of variance (ANOVA) and Tukey’s post hoc test were used to compare the groups. Differences were considered significant at *p* ≤ 0.05.

## 4. Conclusions

To sum up, the gastric digests of adzuki and mung bean sprouts exhibited multidirectional antioxidant activities. Importantly, sprouts obtained in the coculture with the probiotic were more effective. The adzuki bean sprout extracts exerted short-term inhibitory effects on the AGS viability already at low concentrations, exerting dose-independent cytostatic effects on the AGS motility. The cytostatic activity of the compounds from the control adzuki bean sprouts was hardly dose-dependent. In turn, the mung sprout extract exerted rather negligible short-term cytostatic effects on the AGS cells in the range of the concentrations applied. The comparison of the results obtained for the control and probiotic-rich sprouts demonstrated that the coculture enhanced the antimotility properties of the sprouts. The quality and quantity of long-term AGS reactions to the adzuki bean sprout extract were more prominent than those observed in the presence of the mung bean sprout extract. Supplementation of the sprouts with the probiotic considerably reduced their antiproliferative properties, especially when the extracts were administered at high concentrations. The cell nuclei of the AGS cells treated with the high concentrations of the extracts were uniformly round or oval and exhibited the characteristics of apoptosis. In the cells treated with the extract obtained from the probiotic-rich sprouts, an increase in the vinculin level was recorded, which may indicate that these extracts exhibit both cytostatic and cytotoxic activities.

## Figures and Tables

**Table 1:** Individual phenolic content in the fraction obtained after the simulated gastric digestion of the control and probiotic-rich adzuki bean and mung bean sprouts.

Compounds	Identification			Content (µg/g of Flour)	
	Rt	(M − H)*m*/*z*		C	LP
	min	MS	MS/MS	Adzuki bean	
Kaempferol 3.7.4′-*O*-triglucoside	3.63	771	447, 285	4.78 ± 0.24a	5.12 ± 1.08a
Kaempferol 3-*O*-glucosyl-rhamnosyl-glucoside	4.04	755	593, 285	10.48 ± 2.50a	12.00 ± 0.62a
Quercetin 3.4′-*O*-diglucoside	4.12	625	463, 301	26.4 ± 2.50a	22.0 ± 3.6a
Quercetin 3-*O*-glucuronide	4.2	477	301	7.16 ± 1.56a	8.22 ± 1.68a
Kaempferol 3-*O*-rhamnosyl-rhamnosyl-glucoside	4.42	739	577, 301	7.72 ± 1.13a	9.06 ± 1.12b
Quercetin 3-*O*-galactoside 7-*O*-rhamnoside	4.64	609	463, 301	11.0 ± 0.80a	12.16 ± 1.92a
Quercetin 3-*O*-rutinoside (Rutin)	4.76	609	463, 301	31.0 ± 0.2a	25.4 ± 2.6b
Unidentified	4.93	481	263	5.00 ± 0.08a	6.00 ± 0.20b
Kaempferol 3-*O*-rutinoside	5.09	593	431, 285	<LOQ	<LOQ
Quercetin-3-*O*-acetylo-hexoside	5.23	505	463, 301	<LOQ	<LOQ
Unidentified	5.32	575	271	<LOQ	<LOQ
Unidentified	6.66	863	269	3.63 ± 0.19b	1.16 ± 0.23a
Unidentified	6.77	287	136	<LOQ	<LOQ
Total				107 ± 9.3a	102 ± 8.7a
				Mung bean	
Kaempferol 3-*O*-glucoside	4.21	447	285	<LOQ	<LOQ
Kaempferol 3-*O*-galactoside	4.35	447	285	<LOQ	<LOQ
Apigenin 3-*O*-glucoside	4.8	431	269	323 ± 23a	364 ± 10b
Apigenin 3-*O*-rutinoside	5.04	577	269	41.0 ± 11a	59.6 ± 4.0a
Apigenin 3-*O*-acetylo-glucoside	5.49	473	269	23.6 ± 4.6a	30.1 ± 1.0b
Total				388 ± 21a	454 ± 18b

Means (±SD) followed by different letters are significantly different (*n* = 9; *p* ≤ 0.05). C—control sprouts, LP—sprouts enriched with *Lactobacillus plantarum*, and<LOQ—below limit of quantification.

**Table 2:** Antioxidant capacity the fraction obtained after simulated gastric digestion of the control and probiotic-rich adzuki bean sprouts.

	Adzuki Bean		Mung Bean	
	C	LP	C	LP
Ability to quench O_2_^−^ (mg TE/g of flour)	2.56 ± 0.17b	1.35 ± 0.10a	2.63 ± 0.12b	3.13 ± 0.13c
Ability to quench ABTS radicals (mg TE/g of flour)	2.48 ± 0.06b	2.50 ± 0.04b	2.02 ± 0.08a	1.74 ± 0.24a
Ability to chelate metal ions (µg EDTA/g of flour)	4.62 ± 0.23b	4.69 ± 0.16b	3.57 ± 0.21a	4.67 ± 0.15b
Ability to quench OH radicals (mg TE/g of flour)	0.50 ± 0.025a	0.55 ± 0.027a	0.69 ± 0.034b	0.90 ± 0.045c
Reducing potential (mg TE/g of flour)	6.21 ± 0.36c	9.30 ± 0.28d	4.79 ± 0.06a	5.12 ± 0.21b
Inhibition of lipids peroxidation (mg TE/g of flour)	5.32 ± 0.19a	6.01 ± 0.26b	5.23 ± 0.26a	4.80 ± 0.65a
Total antioxidantactivity index	1.00	1.13	1.00	1.09

Means (±SD) for the selected activity followed by different letters are significantly different (*n* = 9; *p* ≤ 0.05). C—control sprouts and LP—sprouts enriched with *Lactobacillus plantarum*. TE—Trolox equivalents.

## References

[1] Bray F., Ferlay J., Soerjomataram I., Siegel R.L., Torre L.A., Jemal A. (2018). Global cancer statistics 2018: GLOBOCAN estimates of incidence and mortality worldwide for 36 cancers in 185 countries. CA Cancer J. Clin..

[2] Golkar L., Ding X.Z., Ujiki M.B., Salabat M.R., Kelly D.L., Scholtens D., Fought A.J., Bentrem D.J., Talamonti M.S., Bell R.H. (2007). Resveratrol inhibits pancreatic cancer cell proliferation through transcriptional induction of macrophage inhibitory cytokine-1. J. Surg. Res..

[3] Roleira F.M.F., Tavares-Da-Silva E.J., Varela C.L., Costa S.C., Silva T., Garrido J., Borges F. (2015). Plant derived and dietary phenolic antioxidants: Anticancer properties. Food Chem..

[4] Gao S., Hu M. (2010). Bioavailability challenges associated with development of anti-cancer phenolics. Mini-Rev. Med. Chem..

[5] Weng C.J., Yen G.C. (2012). Flavonoids, a ubiquitous dietary phenolic subclass, exert extensive in vitro anti-invasive and in vivo anti-metastatic activities. Cancer Metastasis Rev..

[6] Campos-Vega R., Loarca-Piña G., Oomah B.D. (2010). Minor components of pulses and their potential impact on human health. Food Res. Int..

[7] Luo J., Cai W., Wu T., Xu B. (2016). Phytochemical distribution in hull and cotyledon of adzuki bean (*Vigna angularis* L.) and mung bean (*Vigna radiate* L.), and their contribution to antioxidant, anti-inflammatory and anti-diabetic activities. Food Chem..

[8] Zou Y., Chang S.K.C. (2011). Effect of black soybean extract on the suppression of the proliferation of human AGS gastric cancer cells via the induction of apoptosis. J. Agric. Food Chem..

[9] Swieca M., Kordowska-Wiater M., Pytka M., Gawlik-Dziki U., Bochnak J., Baraniak B. (2018). *Lactobacillus plantarum* 299V improves microbiological quality of legume sprouts and effectively survives in those carriers during cold storage and digestion in vitro. PLoS ONE.

[10] Swieca M., Kordowska-Wiater M., Pytka M., Gawlik-Dziki U., Seczyk L., Złotek U., Kapusta I. (2019). Nutritional and pro-health quality of lentil and adzuki bean sprouts enriched with probiotic yeast *Saccharomyces cerevisiae* var. boulardii. LWT Food Sci. Technol..

[11] Zhao K., Xie Q., Xu D., Guo Y., Tao X., Wei H., Wan C. (2018). Antagonistics of *Lactobacillus plantarum* ZDY2013 against Helicobacter pylori SS1 and its infection in vitro in human gastric epithelial AGS cells. J. Biosci. Bioeng..

[12] Hyun H.B., Moon J.Y., Cho S.K. (2018). Quercetin suppresses CYR61-mediated multidrug resistance in human gastric adenocarcinoma AGS cells. Molecules.

[13] Plaza-Díaz J., Ruiz-Ojeda F.J., Vilchez-Padial L.M., Gil A. (2017). Evidence of the anti-inflammatory effects of probiotics and synbiotics in intestinal chronic diseases. Nutrients.

[14] Markowiak P., Śliżewska K. (2017). Effects of probiotics, prebiotics, and synbiotics on human health. Nutrients.

[15] George Kerry R., Patra J.K., Gouda S., Park Y., Shin H.S., Das G. (2018). Benefaction of probiotics for human health: A review. J. Food Drug Anal..

[16] Arena M.P., Silvain A., Normanno G., Grieco F., Drider D., Spano G., Fiocco D. (2016). Use of *Lactobacillus plantarum* strains as a bio-control strategy against food-borne pathogenic microorganisms. Front. Microbiol..

[17] Limón R.I., Peñas E., Martínez-Villaluenga C., Frias J. (2014). Role of elicitation on the health-promoting properties of kidney bean sprouts. LWT Food Sci. Technol..

[18] Luna-Vital D., González de Mejía E. (2018). Peptides from legumes with antigastrointestinal cancer potential: Current evidence for their molecular mechanisms. Curr. Opin. Food Sci..

[19] Ghavidel R.A., Prakash J. (2007). The impact of germination and dehulling on nutrients, antinutrients, in vitro iron and calcium bioavailability and in vitro starch and protein digestibility of some legume seeds. LWT Food Sci. Technol..

[20] Gulewicz P., Martinez-Villaluenga C., Kasprowicz-Potocka M., Frias J. (2014). Non-nutritive compounds in fabaceae family seeds and the improvement of their nutritional quality by traditional processing–A review. Polish J. Food Nutr. Sci..

[21] Zhang Y., Seeram N.P., Lee R., Feng L., Heber D. (2008). Isolation and identification of strawberry phenolics with antioxidant and human cancer cell antiproliferative properties. J. Agric. Food Chem..

[22] Tang D., Dong Y., Guo N., Ren H. (2014). Metabolomic analysis of the polyphenols in germinating mung beans (*Vigna radiata*) seeds and sprouts. J. Sci. Food Agric..

[23] Bai Y., Xu Y., Wang B., Li S., Guo F., Hua H., Zhao Y., Yu Z. (2017). Comparison of phenolic compounds, antioxidant and antidiabetic activities between selected edible beans and their different growth periods leaves. J. Funct. Foods.

[24] Seczyk L., Swieca M., Gawlik-Dziki U. (2017). Soymilk enriched with green coffee phenolics - Antioxidant and nutritional properties in the light of phenolics-food matrix interactions. Food Chem..

[25] Rodríguez-Roque M.J., Rojas-Graü M.A., Elez-Martínez P., Martín-Belloso O. (2013). Soymilk phenolic compounds, isoflavones and antioxidant activity as affected by in vitro gastrointestinal digestion. Food Chem..

[26] Kim S.E., Kim Y.S., Shin W.B., Park J.S., Moon S.H., Jeon B.T., Park P.J. (2018). Induction of caspase-mediated apoptosis using *Alnus japonica* extracts in AGS human gastric carcinoma cells. J. Appl. Biomed..

[27] Chen J., Chen J., Li Z., Liu C., Yin L. (2014). The apoptotic effect of apigenin on human gastric carcinoma cells through mitochondrial signal pathway. Tumor Biol..

[28] Lefort É.C., Blay J. (2013). Apigenin and its impact on gastrointestinal cancers. Mol. Nutr. Food Res..

[29] Wang P., Zhang K., Zhang Q., Mei J., Chen C., Feng Z. (2012). Effects of quercetin on the apoptosis of the human gastric carcinoma cells. Toxicol. Vitr..

[30] Gawande S., Kale A., Kotwal S. (2008). Effect of nutrient mixture and black grapes on the pharmacokinetics of orally administered (-)epigallocatechin-3-gallate from green tea extract: A human study. Phyther. Res..

[31] Xu B., Chang S.K.C. (2012). Comparative study on antiproliferation properties and cellular antioxidant activities of commonly consumed food legumes against nine human cancer cell lines. Food Chem..

[32] Schantz M., Mohn C., Baum M., Richling E. (2010). Antioxidative efficiency of an anthocyanin rich bilberry extract in the human colon tumor cell lines Caco-2 and HT-29. J. Berry Res..

[33] Minekus M., Alminger M., Alvito P., Ballance S., Bohn T., Bourlieu C., Carrì F., Boutrou R., Corredig F.M., Dupont D. (2014). A standardised static in vitro digestion method suitable for food—An international consensus. Food Funct..

[34] Oszmiański J., Wojdyło A., Gorzelany J., Kapusta I. (2011). Identification and characterization of low molecular weight polyphenols in berry leaf extracts by HPLC-DAD and LC-ESI/MS. J. Agric. Food Chem..

[35] Pulido R., Bravo L., Saura-Calixto F. (2000). Antioxidant activity of dietary polyphenols as determined by a modified ferric reducing/antioxidant power assay. J. Agric. Food Chem..

[36] Goupy P., Vulcain E., Caris-Veyrat C., Dangles O. (2007). Dietary antioxidants as inhibitors of the heme-induced peroxidation of linoleic acid: Mechanism of action and synergism. Free Radic. Biol. Med..

[37] Decker E.A., Welch B. (1990). Role of ferritin as a lipid oxidation catalyst in muscle food. J. Agric. Food Chem..

[38] Re R., Pellegrini N., Proteggente A., Pannala A., Yang M., Rice-Evans C. (1999). Antioxidant activity applying an improved ABTS radical cation decolorization assay. Free Radic. Biol. Med..

[39] Su X.Y., Wang Z.Y., Liu J.R. (2009). In vitro and in vivo antioxidant activity of *Pinus koraiensis* seed extract containing phenolic compounds. Food Chem..

[40] Swieca M., Sęczyk L., Gawlik-Dziki U. (2014). Elicitation and precursor feeding as tools for the improvement of the phenolic content and antioxidant activity of lentil sprouts. Food Chem..

[41] Daniel-Wójcik A., Misztal K., Bechyne I., Sroka J., Miekus K., Madeja Z., Czyz J. (2008). Cell motility affects the intensity of gap junctional coupling in prostate carcinoma and melanoma cell populations. Int. J. Oncol..

